# Editorial: Plastid Proteostasis: Relevance of Transcription, Translation, and Post-translational Modifications

**DOI:** 10.3389/fpls.2017.01759

**Published:** 2017-10-17

**Authors:** Fiammetta Alagna, Michele Bellucci, Dario Leister, Andrea Pompa

**Affiliations:** ^1^Viticoltura ed Enologia, Consiglio per la Ricerca in Agricoltura e l'Analisi dell'Economia Agraria (CREA), Turi, Italy; ^2^Istituto di Bioscienze e Biorisorse, Consiglio Nazionale delle Ricerche (CNR), Perugia, Italy; ^3^Plant Molecular Biology, Department Biology I, Ludwig-Maximilians-Universität, München, Germany

**Keywords:** plastome, gene expression, protein balance, regulation, nuclear-plastid interactions

Plastids are sites of biochemical and biological processes that are fundamental for plant life. The genome of these endosymbiotic organelles encodes for almost one hundred of the three thousand proteins that make up the chloroplast proteome. Genes coding for plastid multi-subunit protein complexes derive from both nuclear and plastid genomes, so it is clear that there is the need of a highly integrated coordination between this two subcellular compartments.

The coordination between the nucleus and the plastome takes place at many different levels, including the modulation of nuclear and plastid transcription, RNA processing and translation, post-translational modifications, and protein targeting. In addition, the plastid retains a whole series of mechanisms for the preservation of its protein balance (proteostasis), including proteases and molecular chaperones (Figure 1).

**Figure 1:**
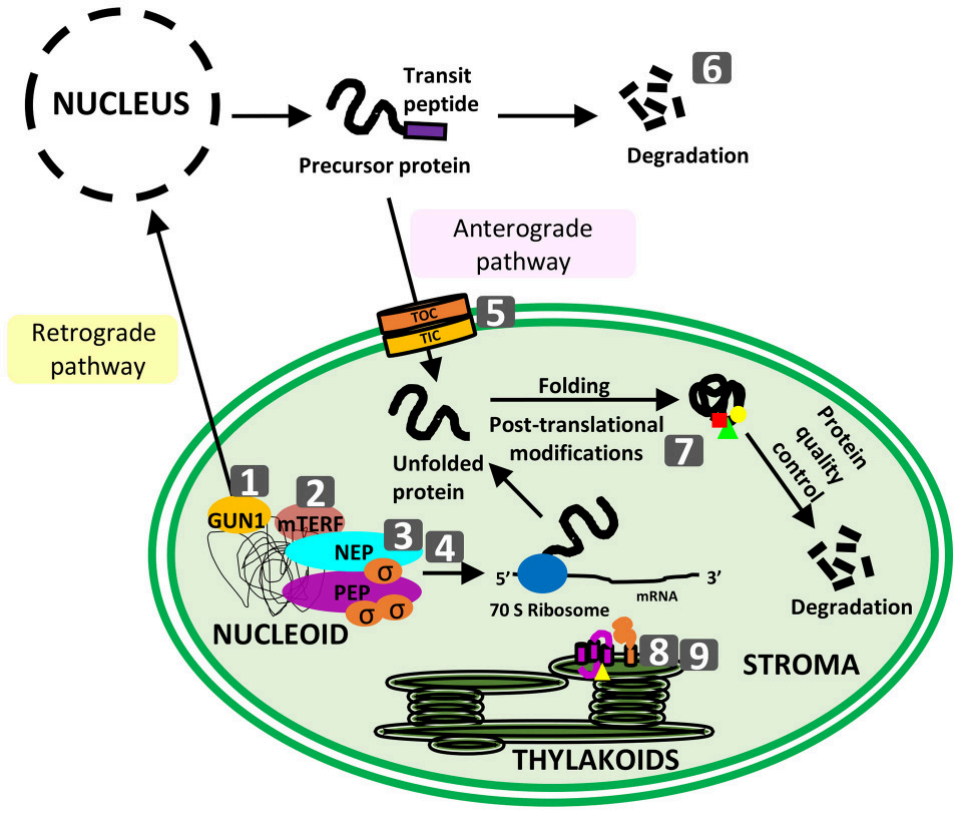
Schematic representation of topics covered in the special issue. Numbers correspond to the following articles: (1) !https://doi.org/10.3389/fpls.2016.01427!!Colombo et al.!!! GUN1, a jack-of-all-trades in chloroplast protein homeostasis and signaling; (2) !https://doi.org/10.3389/fpls.2017.01213!!Xu et al.!!! *Arabidopsis thaliana* mTERF10 and mTERF11, but not mTERF12, are involved in the response to salt stress; (3) !https://doi.org/10.3389/fpls.2017.00023!!Liebers et al.!!! regulatory shifts in plastid transcription play a key role in morphological conversions of plastids during plant development; (4) !https://doi.org/10.3389/fpls.2017.01186!!Shimmura et al.!!! comparative analysis of chloroplast *psbD* promoters in terrestrial plants; (5) !https://doi.org/10.3389/fpls.2017.00168!!Sjuts et al.!!! import of soluble proteins into chloroplasts and potential regulatory mechanisms; (6) !https://doi.org/10.3389/fpls.2017.00310!!Hirosawa et al.!!! ubiquitin-proteasome-dependent regulation of bidirectional communication between plastids and the nucleus; (7) !https://doi.org/10.3389/fpls.2017.00240!!Grabsztunowicz et al.!!! post-translational modifications in regulation of chloroplast function: recent advances; (8) !https://doi.org/10.3389/fpls.2017.01313!!Gabilly and Hamel!!!, maturation of plastid *c*-type cytochromes; (9) !https://doi.org/10.3389/fpls.2017.01306!!Cline et al.!!! CCS2, an octatricopeptide-repeat protein, is required for plastid cytochrome *c* assembly in the green alga *Chlamydomonas reinhardtii*.

Plastids have largely abandoned transcriptional control switching predominantly to translational and post-translational control of their gene expression, but some transcriptional regulation is known to occur. Transcription of plastid genes is performed by two different types of RNA polymerases: plastid-encoded RNA polymerase (PEP) and a nuclear-encoded RNA polymerases (NEP). !https://doi.org/10.3389/fpls.2017.00023!!Liebers et al.!!! propose that targeted changes in plastid transcription, mostly by controlling the relative activities of NEP and PEP enzymes, impact the establishment of the plastid proteome and these represent key determinants for the transitions between the different plastid types.

The transcriptional regulation mechanisms are still far from being completely elucidated. An unusual light- and stress-responsive promoter (*psbD* LRP), regulated by a AAG-box immediately upstream of the –35 element, has been recently mapped. !https://doi.org/10.3389/fpls.2017.01186!!Shimmura et al.!!! analyzed *psbD* LRP promoter regions in 11 embryophytes, at different evolutionary stages, from liverworts to angiosperms. This analysis identified conserved features of the promoter and facilitated study of its emergence and evolution in plant species.

Among the proteins that regulate plastid gene expression, the nucleus-encoded proteins of the mitochondrial transcription termination factor (mTERF) family have been recently identified. Information on their function is only beginning to emerge. !https://doi.org/10.3389/fpls.2017.01213!!Xu et al.!!! investigate the function of the chloroplast-associated mTERF. They report that these proteins are localized to chloroplast nucleoids and identify two of them involved in the salt stress response.

The import of plastid precursor proteins into plastids is another checkpoint affecting plastid proteostasis that is regulated in response to the fluctuating environmental conditions. This fine regulation ensures the optimal functioning of important biological processes taking place in this cellular compartment. The import of plastid precursor proteins is mediated by two distinct translocation complexes called TOC and TIC, located respectively at the outer and at the inner envelope membrane of chloroplasts. The individual steps involved in protein translocation and the corresponding regulation mechanisms used by plants to modulate protein import are reviewed by !https://doi.org/10.3389/fpls.2017.00168!!Sjuts et al.!!!

Upon transition from an endosymbiont to a plant cell organelle, the plastid retains a set of mechanisms that involves enzymes and proteins of prokaryotic origin which are responsible for protein maturation, post-translational modification, correct folding, protein abundance control, and removal of misfolded or damaged components. These processes ensure that plastid proteins are ready to exert their biological mission and require an intricate system of signals from nucleus to plastid and backwards. Plastid-derived signals can regulate availability of nuclear-encoded plastid precursors controlling their *de novo* synthesis, and targeting. Recently, an important player in the chloroplast-to-nucleus retrograde communication has been identified: the protein GENOMES UNCOUPLED1 (GUN1). Recent studies indicate that GUN1 might play a role in the coordination of translation, import, and degradation of plastid proteins. The molecular function of different GUN1 partners has been reviewed by !https://doi.org/10.3389/fpls.2016.01427!!Colombo et al.!!!, highlighting its potential role in plastid proteostasis. Another important mechanism during nuclear-plastid interaction is the degradation of multiple components through the ubiquitin–proteasome system. It has become increasingly clear that, together with feedback regulation of nuclear gene expression by plastid-derived signals, this mechanism avoids the accumulation of non-imported proteins in the cytosol. In addition to the anterograde signaling pathway, recent studies in *A. thaliana* demonstrated that also the retrograde signaling pathway can be subjected to ubiquitin–proteasome regulation. !https://doi.org/10.3389/fpls.2017.00310!!Hirosawa et al.!!! review recent advances in understanding how the ubiquitin–proteasome system regulates the nuclear–plastid interaction and plastid biogenesis.

The maturation of plastid proteins is another highly regulated process that in some cases needs complex apparatuses to occur. This is the case of *c*-type cytochromes that require a multicomponent assembly pathway for their maturation, as reviewed by !https://doi.org/10.3389/fpls.2017.01313!!Gabilly and Hamel!!!. The regulation of this pathway has not yet been clarified. !https://doi.org/10.3389/fpls.2017.01306!!Cline et al.!!! describe the functional characterization of the *CCS2* gene of *Chlamydomonas reinhardtii* required for cytochrome *c* assembly. The authors discuss the possible functions of CCS2 in the heme attachment reaction.

A wide range of post-translational modifications (PTMs) contribute to finely regulate the biological processes that take place in plastids. PTMs alter the physicochemical properties of the plastidial proteins thus affecting their function. !https://doi.org/10.3389/fpls.2017.00240!!Grabsztunowicz et al.!!! review the current knowledge on the PTMs regulating important metabolic processes in chloroplasts such as DNA replication and gene expression, photosynthetic carbon assimilation, and starch metabolism and report the known physiological effects of these modifications.

Considering that there is limited knowledge of the combined action among the molecular mechanisms that regulate plastid proteostasis, the goal of this Research Topic is to bring together a set of articles that contribute to our understanding of how transcription, translation, and post-translational modifications (including protein targeting) maintain plastid proteostasis. As a consequence, this Research Topic will help us understand more deeply how plastids function.

## Author contributions

MB, DL, and AP prepared the research topic. All the authors edited the manuscripts. FA invited the authors and wrote the editorial. All the authors read and approved the editorial.

### Conflict of interest statement

The authors declare that the research was conducted in the absence of any commercial or financial relationships that could be construed as a potential conflict of interest.

